# Digital Wellness Programs in the Workplace: Meta-Review

**DOI:** 10.2196/70982

**Published:** 2025-03-14

**Authors:** Saeed Amirabdolahian, Guy Pare, Stefan Tams

**Affiliations:** 1 Information Technology Department HEC Montréal Montreal, QC Canada; 2 Research Chair in Digital Health HEC Montréal Montreal, QC Canada

**Keywords:** digital wellness programs, corporate wellness, health interventions, efficacy, acceptability, meta-review, mHealth, eHealth

## Abstract

**Background:**

Corporate wellness programs are increasingly using digital technologies to promote employee health. Digital wellness programs (DWPs) refer to initiatives that deliver health interventions through digital tools. Despite a growing body of evidence on DWPs, the literature remains fragmented across multiple health domains.

**Objective:**

This study aims to provide a comprehensive synthesis of existing research on the efficacy (eg, impact on employee’s physical health, mental well-being, behavioral changes, and absenteeism) and acceptability (eg, engagement, perceived usefulness, and adoption) of employer-provided DWPs. Specifically, we aim to map the extent, range, and nature of research on this topic; summarize key findings; identify gaps; and facilitate knowledge dissemination.

**Methods:**

We conducted a meta-review of studies published between 2000 and 2023. We adopted a database-driven search approach, including the MEDLINE, PsycINFO, ProQuest Central, and Web of Science Core Collection databases. The inclusion criteria consisted of (1) review articles; (2) publications in English, French, or German; (3) studies reporting on digital health interventions implemented in organizations; (4) studies reporting on nonclinical or preclinical employee populations; and (5) studies assessing the efficacy and acceptability of employer-provided DWPs. We performed a descriptive numerical summary and thematic analysis of the included studies.

**Results:**

Out of 593 nonduplicate studies screened, 29 met the inclusion criteria. The most investigated health domains included mental health (n=19), physical activity (n=8), weight management (n=6), unhealthy behavior change (n=4), and sleep management (n=2). In total, 24 reviews focused on the efficacy of DWPs, primarily in relation to health-related outcomes (eg, stress and weight), while fewer reviews addressed organization-related outcomes (eg, burnout and absenteeism). Four reviews explored the mechanisms of action, and 3 assessed the acceptability of DWPs using various measures. Overall, the findings support the efficacy and acceptability of DWPs, although significant gaps persist, particularly regarding the durability of outcomes, the role of technology, and the causal mechanisms underlying behavioral change.

**Conclusions:**

While DWPs show promise across a variety of health domains, several aspects of their effectiveness remain underexplored. Practitioners should capitalize on existing evidence of successful DWPs while acknowledging the limitations in the literature.

## Introduction

Employee health is closely intertwined with stakeholders’ financial interests, and technology can play a pivotal role in advancing both. A recent large-scale survey identified a clear link between employee well-being and firm performance [1]. Beyond this alignment, employee wellness has become an organizational expectation [2], with surveys indicating that most employees believe their organizations are responsible for supporting their health and well-being [3,4]. To promote employee health, many companies invest in corporate wellness programs, with more than half of the organizations offering at least one such initiative [5]. These programs have evolved into a strategic priority, not only as an attractive job benefit but also as a means to reduce absenteeism and lower health care costs [6].

A meta-analysis found that for every one US dollar invested in corporate wellness programs, organizations save US $3.27 in health care costs and US $2.73 through reduced absenteeism [7]. Beyond financial benefits, these programs foster a sense of organizational support, enhancing employee commitment and prosocial behavior in the workplace [8-10]. In addition, the growing emphasis on corporate social responsibility has drawn practitioners’ attention to wellness programs as a means to fulfill these expectations [11]. Given the significant amount of time employees spend at work, the workplace provides a unique environment for implementing wellness initiatives. Organizations can effectively scale these programs by identifying key risk factors and integrating targeted wellness initiatives into their routines and culture.

The growing integration of digital technologies into wellness programs has significantly improved their accessibility and scalability. However, despite the expanding body of research on digital wellness programs (DWPs), the literature remains highly fragmented across multiple domains and disciplines. This fragmentation poses challenges for organizational practitioners, making it difficult to adopt evidence-based best practices effectively.

In this study, we define DWPs as initiatives that deliver intervention content to employees through digital tools, either partially or fully. DWPs are not simply digital updates of traditional programs; they exhibit unique features that merit focused research for several reasons. First, participants in DWPs play a more active role than in traditional wellness programs. They can creatively adapt and personalize digital tools, which can influence the programs’ outcomes [12]. Second, digital technologies add complexity to the design of wellness programs. Previous studies have highlighted factors such as interaction modularity [13], the design of digital nudges [14], and engagement with technology features [15] as critical determinants of the efficacy of DWPs. Third, digital technologies enable novel ways of interacting with employees, creating innovative wellness solutions previously unattainable. For example, automated digital interventions can provide health feedback without direct human supervision. These considerations have spurred a growing interest in research on DWPs.

Despite the considerable body of research, knowledge on DWPs is still fragmented across multiple disciplines. This fragmentation is reflected in the numerous reviews that examine the same topic yet reach varying conclusions. The effectiveness of DWPs often depends on how their boundaries are defined. For instance, some reviews report highly promising outcomes (eg, [16,17]), while others highlight conflicting evidence, raising doubts about their overall efficacy (eg, [18,19]). This poses a challenge, as review studies play a critical role in guiding practitioners’ decision-making [20,21]. In addition, research on DWPs is expanding rapidly across various health domains (eg, mental health, physical activity, and weight management) yet often develops in silos, limiting the generalizability of findings beyond specific areas. This fragmentation is particularly concerning because, regardless of the health domain, DWPs share key similarities in their behavioral mechanisms, technological components, and target populations. To address this gap, this study provides a comprehensive overview of the literature on DWPs, aiming to navigate the scattered research landscape and facilitate cross-pollination of ideas to inform future research.

Given the abundance of studies and reviews on DWPs, we have adopted a meta-review methodology to cover the broad scope of the relevant literature. This approach ensures that we address all compelling evidence and identify research gaps in the field. This meta-review began with the broad research questions (RQs): “What do we know?” and “What remains unanswered?” During the full-text screening process, it became clear that the central focus of the literature revolves around the efficacy and acceptability of DWPs. Thus, the initial questions were refined into the following specific research inquiries:

1. What technologies and health domains have been investigated in prior research on DWPs?
2. What do we know about the efficacy and acceptability of DWPs?

This meta-review answers these questions by categorizing the existing literature into 6 health domains identified inductively. This classification helps to uncover avenues for future research within each domain, identify points of agreement and disagreement, compare findings across domains, and encourage the cross-pollination of ideas.

In short, the primary objective of this study is to synthesize existing research on the efficacy and acceptability of DWPs in workplace settings. Specifically, we aim to (1) assess the effectiveness of DWPs in improving employees’ physical and mental well-being, (2) examine the acceptability and engagement levels of these interventions, and (3) identify gaps in the literature to inform future research and practice.

## Methods

### Research Design

This study uses a meta-review method, which is particularly useful in interdisciplinary research when an extensive body of literature exists, and a broad exploration of the topic is necessary [22]. This approach uses various types of literature reviews as input and adopts a comprehensive search strategy and considers diverse literature to address broad RQs [23,24].

The meta-review method aligns well with our research objectives, considering that an initial exploratory search revealed that synthesizing existing reviews would be valuable. Much of the relevant knowledge is dispersed across various review studies on closely related topics, yet many of these reviews were conducted independently, without referencing findings from other reviews. This lack of integration limits the potential to consolidate insights across similar reviews.

As protocol registration is not a standard requirement for meta-reviews, this study was not prospectively registered. However, to ensure transparency and systematicity [25,26], we adhered to the PRISMA (Preferred Reporting Items for Systematic Reviews and Meta-Analyses) framework (Multimedia Appendix 1 [27]) and followed the guidelines set forth by Paré et al [25].

### Search Strategy

The search process followed 3 key steps, as recommended by Gusenbauer and Haddaway [28]. The first step involved a preliminary lookup search on Google Scholar to identify existing reviews on DWPs. This step allowed us to assess the feasibility of conducting a scoping meta-review and helped pinpoint key pieces of evidence on the topic.

The second step was an exploratory search to determine the approximate scope of the topic. The authors carried out the search with the assistance of a professional librarian and discussed initial findings to develop a comprehensive list of search terms and refine the search queries accordingly.

Finally, the third step involved a systematic search of relevant studies published between January 2000 and December 2023. The search was initially carried out in July 2023 and updated in January 2024. The search timeframe (2000-2023) was chosen to capture the evolution of DWPs in the workplace. Digital components of wellness programs began gaining traction in the early 2000s, and review articles synthesizing research in this area started appearing around this time. This timeframe ensures comprehensive coverage of relevant literature while maintaining a focused and manageable scope. We adopted a database-driven search approach [29], using 4 multidisciplinary research databases: MEDLINE, PsycINFO, ProQuest Central and the Web of Science Core Collection. This multistep process ensured that the search terms and databases used captured the breadth of this multidisciplinary topic. In addition, we conducted a forward snowballing search to complement the systematic search.

Textbox 1 presents the finalized search terms and the logical structure of the search query. Since we aimed to capture all ranges of intervention outcomes and comparisons, the search query did not include them. The search string used in each database can be found in Multimedia Appendix 2.

**Search terms and the logical combinations of conducted queries.**

*(systematic review OR scoping review OR narrative review OR descriptive review OR meta-analysis OR mapping review OR realist review OR meta-synthesis OR comparative review OR conceptual review OR literature review OR integrative review OR meta-ethnography OR meta-synthesis OR meta-review OR narrative review OR narrative synthesis OR qualitative review)*

*AND*

*(workplace OR work place OR worker OR staff OR workforce OR employee OR occupational setting OR organizational setting OR organisational setting)*

*AND*

*(m-health OR mhealth OR mobile health OR e-health OR ehealth OR digital health OR digital wellness OR virtual care OR self-tracking OR self-quantification OR quantified-self OR wearable OR iCBT) OR ((physical health OR physical activity OR mental health OR sleep health OR psychological health OR well-being OR wellbeing OR prescriptions OR preventive health OR sleep health OR sleep disorders) AND (app OR information technology OR information system OR digital OR platform OR self-quantification OR quantified self OR smartphone OR internet-based OR mobile phone OR cell phone OR virtual OR teleconsultation))
*

### Screening

A set of inclusion and exclusion criteria was developed to clearly define the scope of this review (Textbox 2). We included all reviews that reported on DWPs aimed at nonclinical or preclinical employee populations. Consistent with our definition of DWPs, interventions were considered digital if their content was delivered to employees fully or partially through digital technologies. The studies had to be available in English, French, or German. We included studies published between January 2000 and the end of 2023, which is a broad timeframe for the subject matter. However, we did not impose a date limit on the primary studies included in the review articles.

When screening literature reviews for inclusion in a meta-review, it is possible that some studies only partially meet the eligibility criteria. For instance, a review might include both digital (eligible) and nondigital (ineligible) interventions. In such cases, we only retained those reviews where the eligible part of the study was analyzed separately.

The identified studies were systematically screened in 2 steps using Covidence, a web-based platform designed for managing and screening citations collaboratively. In the first step, one reviewer screened the abstracts of the identified studies and excluded only those that clearly did not meet the inclusion criteria. Where there was uncertainty, the paper was flagged for further discussion. Flagged papers were then reviewed in working sessions where the authors reached a consensus before making any exclusion decisions. Papers not excluded at this stage were retained for full-text screening. In the second step, 3 independent reviewers conducted the full-text screening. Each reviewer read and assessed the papers independently. In 2 subsequent working sessions, any disagreements were resolved through discussion, ensuring a final consensus on the inclusion of each paper.

**List of inclusion and exclusion criteria.**

**Inclusion criteria**

- Population
  Any nonclinical or preclinical working population
  Studies that focused on working and nonworking populations simultaneously were included only if they present their findings regarding the working population separately
- Intervention and exposure
  Any digital health interventions used in workplace settings. It includes interventions aimed at employees’ physical health; mental health; well-being; prescriptions; or preventive health, employee assistance programs, substance or alcohol misuse interventions, sexual health interventions, sleep disorders, etc
  Studies that have focused on digital and nondigital health interventions were included only if they have presented their findings regarding the working population separately
- Outcomes
  Efficacy or acceptability outcomes
- Study design
  Stand-alone review articles in peer-reviewed outlets
  Review reports from governments, industry leaders, and prominent consulting firms
- Time
  Published between January 2000 and December 2023

**Exclusion criteria**

- Population
  Any population outside of a workplace setting (eg, students)
  Special occupational groups without typical freedoms (eg, soldiers) or with informal employment (eg, sex workers)
- Intervention and exposure
  Digital health interventions not supported or funded by workplaces, transition-to-retirement interventions, and interventions aimed at clinical populations (eg, patients with cancer)
  Interventions mainly based on outdated digital technologies such as CD-ROMs and PDAs were excluded
  Interventions specifically implemented during the pandemic
  Long-term management of mental illnesses
- Outcomes
  No exclusion
- Study design
  Primary studies
  Review protocols
  Conference abstract
  Theses and dissertations
- Time
  No exclusion

### Data Charting

In this stage, we charted key information from the included studies. We adapted an existing data extraction tool designed for meta-review studies [30]. This tool includes 17 data items: title, author or authors, year of publication, place of publication, research objectives, review type, information sources searched, inclusion years range, inclusion and exclusion criteria, search terms used, corpus size, aim of interventions, reported work and health outcomes, geographic dispersion of the included studies, organizational settings, intervention characteristics, intervention durations, and key findings. Microsoft Excel was used to chart the data for analysis. As recommended by review methodology guidelines, this process was highly iterative [31]. One author initially extracted the data items for all included studies. Subsequently, all authors participated in 4 working sessions to discuss and validate the data extraction table. The analysis of the extracted data is presented in the following section, and the full dataset is available upon request from the first author.

### Data Analysis

The data analysis comprised a descriptive numerical summary and a thematic analysis [31]. The descriptive numerical summary involved a straightforward process of characterizing the included studies. However, the thematic analysis used a highly iterative process. Specifically, we applied conventional qualitative content analysis, which is appropriate when no prior theoretical framework exists and researchers aim to avoid using preconceived categories [32]. Although there are several behavior change theories for various health interventions, no overarching framework or theory is available for organizing the diverse interventions within DWPs across different health domains.

Following the approach outlined by Hsieh and Shannon [32], themes were allowed to emerge naturally from the data through an iterative process. This involved carefully reading the data items, deriving codes, making notes on the emerging codes, revisiting the texts to contextualize the codes, and refining the coding scheme to develop themes. This process unfolded over the course of 6 working sessions, during which the emerging themes were discussed and refined. The result of this step was the categorization of health domains, RQs, and digital technologies and the separation of evidence on efficacy outcomes from mechanisms of action. The Results and Discussion sections are structured according to these categories.

## Results

### Summary of the Search and Screening Process

**Figure 1:**
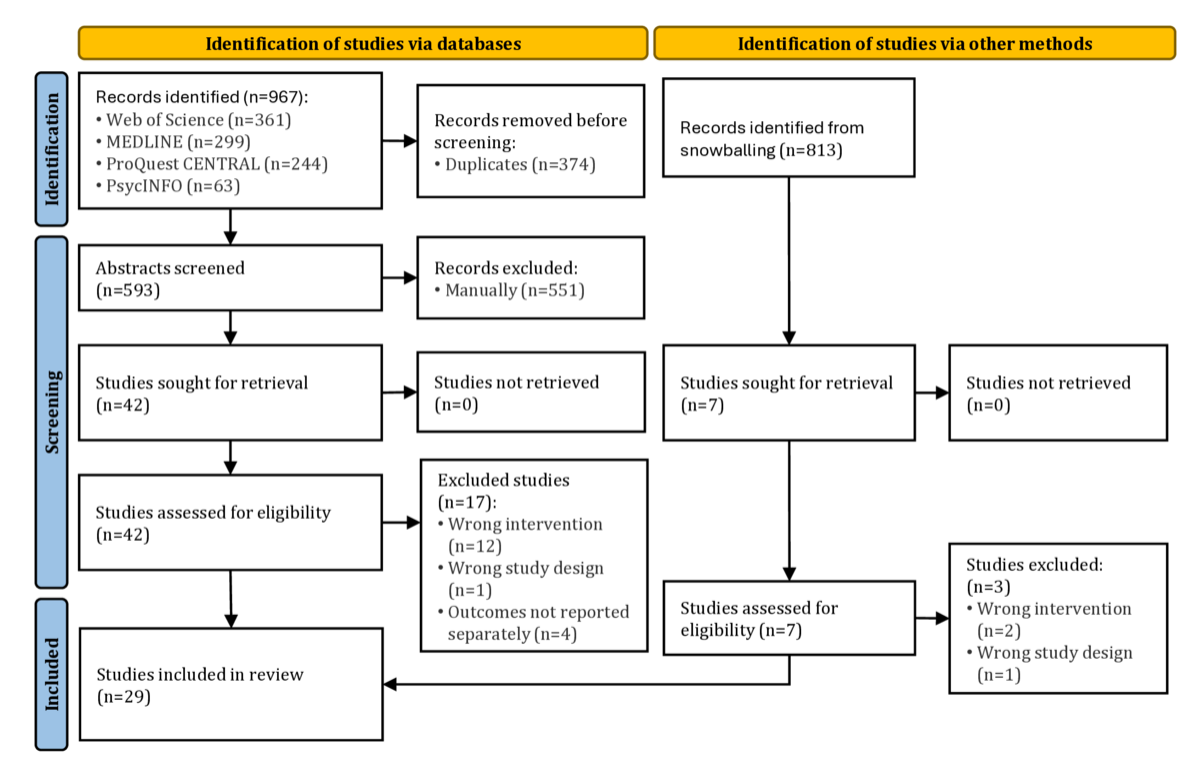
PRISMA (Preferred Reporting Items for Systematic Reviews and Meta-Analyses) flowchart.

The PRISMA flowchart shown in Figure 1 outlines the search and screening process. Initially, the search yielded 967 records, from which 374 duplicates were removed. This left 593 studies, which were screened by abstract. After this step, 42 studies were identified as eligible for full-text screening. The authors ultimately agreed to include 25 studies in the corpus. To ensure thoroughness, a snowballing search was conducted, retrieving a list of 813 papers from Google Scholar using web-scraping tools. One author screened the titles and abstracts for potentially relevant papers which were manually retrieved for full-text review. This process added 7 papers for full-text screening, of which 4 were included in the final corpus. As a result, the search and screening process concluded with a total of 29 studies included in the database.

### Characteristics of the Included Studies

This section outlines the key characteristics of the included studies.

#### Date of Publication

Although our inclusion criteria limited the selection to studies published since 2000, the earliest identified review article appeared in 2014, confirming the appropriateness of our chosen publication period. Notably, there was a significant surge in publications in 2023, with 9 out of the 11 studies from that year focusing specifically on DWPs aimed at improving employees’ mental health. Unsurprisingly, all these studies cited the recent COVID-19 pandemic as a key motivation.

#### Health Domains

As shown in Table 1, DWP interventions concern 6 health domains. Mental health (n=19) emerged as the most frequently investigated domain, encompassing interventions aimed at addressing employees’ stress, anxiety, burnout, and overall quality of life. The next most explored domains are physical activities (n=8), weight management (n=6), unhealthy behavior change (n=4), and sleep management (n=2). Four studies were categorized as “other.” These include programs aimed at screening and reducing varied health risk factors that relate to diabetes and other chronic diseases.

**Table 1:** Profile of included studies.

Study	Search range	Review type	Corpus size	Type of research question	Health domains^a-h^					
					Mental health^a^	Physical activity^b^	Weight management^c^	Unhealthy behavior change^d^	Sleep management^e^	Others^f^
Aneni et al [18]	Up to and including 2012	Systematic review (narrative analysis)	29	Efficacy		✓	✓	✓		
Armaou et al [33]	1990-2019	Systematic review (narrative analysis)	51	Efficacy	✓					
Bégin et al [34]	Up to and including 2021	Scoping review	56	Efficacy	✓					
Buckingham et al [35]	2007-2017	Systematic review (narrative analysis)	25	Efficacy, mechanisms of action, acceptability		✓				
Bullock et al [19]	2009-2021	Scoping review	2	Efficacy					✓	
Carolan et al [36]	2000-2016	Meta-analysis	21	Efficacy, acceptability	✓					
Damen et al [37]	2009-2019	Scoping review	45	Mechanisms of action		✓				
Freak-Poli et al [38]	Up to and including 2016	Meta-analysis	14	Efficacy		✓				
Jones et al [39]	2014-2019	Systematic review (narrative analysis)	12	Efficacy	✓					
Jung and Cho [40]	Up to and including 2020	Meta-analysis	8	Efficacy		✓	✓			
Kuster et al [41]	Up to and including 2017	Systematic review (narrative analysis)	2	Efficacy						
Lee et al [42]	Up to and including 2021	Systematic review (narrative analysis)	11	Efficacy			✓			
López-Del-Hoyo et al [43]	Up to and including 2022	Systematic review (narrative analysis)	27	Efficacy	✓					
Moe-Byrne et al [44]	2000-2022	Systematic review (narrative analysis)	7	Efficacy	✓					
Paganin and Simbula [45]	2009-2019	Systematic review (narrative analysis)	31	Efficacy	✓		✓			✓
Park et al [46]	Up to and including 2021	Systematic review (narrative analysis)	7	Efficacy	✓					
Phillips et al [16]	Up to and including 2018	Meta-analysis	50	Efficacy	✓			✓		
Scheutzow et al [47]	2005-2019	Systematic review (narrative analysis)	28	Acceptability	✓					
Sevic et al [48]	1990-2021	Systematic review (narrative analysis)	17	Efficacy, mechanisms of action		✓		✓		✓
Stratton et al [49]	1975-2016	Meta-analysis	23	Efficacy	✓					
Stratton et al [50]	1991-2019	Meta-analysis	28	Efficacy	✓					
Stratton et al [17]	2004-2020	Meta-analysis	75	Efficacy	✓					
Sundstrom et al [51]	2000-2021	Scoping review	20	Efficacy				✓		
Thai et al [52]^g^	2010-2021	Scoping review	24	Efficacy	✓	✓	✓		✓	✓
Tung et al [53]	2006-2020	Systematic review (narrative analysis)	14	Mechanisms of action		✓	✓			✓
Velana and Rinkenauer [54]	2000-2020	Systematic review (narrative analysis)	6^h^	Efficacy	✓					
Vertola et al [55]	2012-2022	Systematic review (narrative analysis)	11	Efficacy	✓					
Webster et al [56]	2010-2018	Scoping review	11	Efficacy	✓					
Xiong et al [57]	Up to and including 2021	Meta-analysis	19	Efficacy	✓					

^a^n=19.

^b^n=8.

^c^n=6.

^d^n=4.

^e^n=2.

^f^n=4.

^g^The inclusion criteria are limited to digital wellness programs in low- and middle-income countries.

^h^The corpus size does not include studies on non-IT interventions.

#### Type of RQs

In line with the objective of this meta-review, we categorized the literature reviews in our corpus into 2 groups based on the RQs they addressed. As shown in Textbox 2, the vast majority of reviews (27/29, 93%) focused on questions related to the efficacy of DWPs. In contrast, only 3 reviews addressed questions concerning the acceptability of these programs, examining criteria such as uptake, continued use, and compliance with the interventions. While efficacy and acceptability were predefined codes, a third category of RQs emerged inductively during the data charting process. Specifically, we identified 4 reviews exploring “how” DWPs influence intervention outcomes. These RQs were coded under interventions' mechanisms of action. Studies in this category primarily examined the behavior change techniques used in interventions and the theoretical explanations of how these interventions drive outcomes.

#### Types of Reviews

We categorized the identified studies according to review types as specified by their authors. In those cases where review types were not explicitly mentioned in a study (eg, [39]), we coded the paper according to the guidelines for review typologies [58].

As shown in Table 1, we identified 3 types of literature reviews in our corpus. The most frequent type was the systematic review, comprising 15 studies. These reviews focus on narrow RQs related to the efficacy of DWPs and typically use narrative synthesis methods rather than statistical techniques to analyze their findings. Similar to meta-analyses, systematic reviews limit their inclusion criteria to experimental and quasi-experimental studies. The second frequent group consisted of 8 meta-analyses, which apply statistical methods to address focused RQs about the efficacy of DWPs. These reviews include experimental and quasi-experimental studies while excluding other empirical research designs. Finally, the least frequent type was the scoping review, with 6 studies. Scoping reviews investigate broader RQs, such as exploring outcomes, theories, and behavior change techniques related to specific groups of DWPs. These reviews aim to provide a comprehensive overview of the existing literature by summarizing a wide range of available evidence.

#### Digital Technologies

The reviews discussing DWPs include various technological components ranging from messaging apps to website portals and telecommunication solutions. We categorized these technologies into five groups: (1) websites and online telecommunication, (2) apps and computer software, (3) email, (4) text messaging services—SMS and messaging apps—and (5) wearable devices. The types of technologies used in each health domain are presented in the mapping tables corresponding to each domain.

### Main Findings

In this section, we present the findings organized according to the 6 health domains discussed earlier.

#### Mental Health

##### Overview

As mentioned earlier, mental health is the most frequently discussed domain in the literature on DWPs. The World Health Organization defines mental health as a state of mental well-being that enables people to cope with the stresses of life, realize their abilities, learn well and work well, and contribute to their community [59]. Anxiety, depression, and stress-related conditions are the most common mental health issues reported in the workforce. Many organizations are interested in mental health as it is the leading cause of disabilities, sick leave requests, and long-term work incapacities [60]. In addition, research shows that improving organizational mental wellness programs can improve productivity and presenteeism at work [61,62]. The identified reviews on mental health DWPs discuss the efficacy and the acceptability of interventions. However, no review was found that examines the mechanisms of action used in this health domain.

Table 2 maps the reviews on mental health DWPs according to the type of RQs asked, the types of digital technologies included, and 12 categories of outcomes reported in the literature. Most reviews in this domain (16/19, 84%) investigate the efficacy of DWPs and include a wide range of digital technologies and intervention outcomes. Except for 4 reviews that are limited to health care occupations [43,46,54,56], all the included reviews have no limitation on the inclusion of working populations.

**Table 2:** Mapping of reviews on mental health digital wellness programs.

Study	Type of RQ^a^	Digital technologies						Outcomes											
		Wb^b^	Ap^c^	Email	Ms^d^	Wearables	Stress		Anxiety	Depression	Sc^e^	Pw^f^	Insomnia	Resilience	En^g^	Absenteeism	Presenteeism	Productivity	Burnout
Stratton et al [49]	Efficacy	✓	✓				✓		✓	✓	✓								
Stratton et al [50]	Efficacy	✓	✓												✓	✓	✓	✓	
Stratton et al [17]	Efficacy	✓	✓				✓		✓	✓									
Armaou et al [33]	Efficacy	✓	✓			✓	✓		✓	✓	✓								
Carolan et al [36]	Efficacy and acceptability	✓	✓	✓	✓							✓						✓	
Xiong et al [57]	Efficacy	✓	✓							✓									
Phillips et al [16]	Efficacy	✓	✓	✓	✓		✓		✓	✓	✓	✓	✓						✓
Kuster et al [41]	Efficacy	✓	✓				✓												
Begin et al [34]	Efficacy	✓	✓			✓	✓		✓	✓	✓			✓					
Jones et al [39]	Efficacy		✓				✓		✓			✓							
Vertola et al [55]	Efficacy		✓				✓					✓							✓
Paganin and Simbula [45]	Efficacy		✓				✓					✓	✓						
Thai et al [52]	Efficacy	✓		✓			✓		✓	✓									
López-Del-Hoyo et al [43]	Efficacy	✓	✓				✓		✓	✓	✓			✓					✓
Moe-Byrne et al [44]	Efficacy	✓	✓	✓			✓		✓	✓			✓	✓			✓		
Park et al [46]	Efficacy	✓	✓				✓		✓	✓				✓					✓
Velana and Rinkenauer [54]	Efficacy	✓	✓				✓		✓	✓									
Webster et al [56]	Efficacy	✓	✓		✓		✓		✓	✓									✓
Scheutzow et al [47]	Acceptability	✓	✓																

^a^RQ: research question.

^b^Wb: websites and online telecommunication.

^c^Ap: apps and software.

^d^Ms: messaging services.

^e^Sc: self-compassion and mindfulness.

^f^Pw: psychological well-being.

^g^En: employee engagement.

##### Efficacy

In the domain of mental health, 17 literature reviews assessed the efficacy of DWPs. Table 3 summarizes the findings from these reviews, grouping the outcomes into 12 different categories. The wide range of efficacy outcomes in this health domain reflects the diversity of programs such as those that aim at reducing stress, anxiety, and depression as well as those that aim at enhancing overall mental wellness among employees. The following paragraphs summarize the findings according to the health outcomes of DWPs.

In some cases, the findings were pooled together, making it difficult to categorize them according to specific mental health outcomes. For instance, a meta-analysis [49] combined outcomes related to stress, anxiety, depression, and mindfulness under the broader term “mental health conditions.” This meta-analysis found a small effect size (*g*=0.24, 95% CI 0.13-0.35, *k*=23, N=1328, *I*²=67%). Subsequent studies have evaluated these results as promising but noted that further clarification is needed regarding specific outcomes [16,17]. In addition, 4 literature reviews [33,34,45,56] did not synthesize or aggregate their findings around specific outcome measures. These reviews primarily conducted scoping surveys of the variability in outcomes and intervention characteristics. The following paragraphs summarize the results of both meta-analyses and non–meta-analyses according to each outcome category.

Stress is the most frequently reported outcome, with 10 reviews addressing its measurement. Two meta-analyses [16,17] evaluated the effectiveness of DWPs on stress. Phillips et al [16] reported a medium-sized effect of DWPs on stress reduction (*g*=0.54, 95% CI 0.35-0.72, *k*=22, N=not available, *I*²=84%). However, Stratton et al [17], using a much larger corpus, reported a smaller effect size for DWPs on stress (*g*=0.25, 95% CI 0.17-0.34, *k*=57, N=10,160, *I*²=76%). Three non–meta-analyses [52,54,55] found unanimous evidence supporting the significant effect of DWPs in reducing stress. However, 5 non–meta-analyses [39,41,43,44,46] reported conflicting findings.

Anxiety is the second most frequently discussed outcome, addressed in 9 reviews. Two meta-analyses [16,17] found small but significant effects of DWPs on anxiety reduction. Stratton et al [17] reported an effect size of *g*=0.26 (95% CI 0.13-0.39, *k*=29, N=4961, *I*²=77%), while Phillips et al [16] found a slightly larger effect size of *g*=0.34 (95% CI 0.18-0.50, *k*=15, N=not available, *I*²=71%). Among non–meta-analyses, 3 reviews [39,52,54] provided unanimous evidence supporting the efficacy of DWPs in reducing anxiety. However, López-Del-Hoyo et al [43] found conflicting evidence, and Park et al [46] reported no significant effect of DWPs on anxiety in the literature.

**Table 3:** Summary of mental health digital wellness program outcomes.

Study	Outcomes											
	Stress	Anxiety	Depression	Sc^a^	Pw^b^	Insomnia	Resilience	En^c^	Absenteeism	Presenteeism	Productivity	Burnout
Stratton et al [49]^d^	s+^e^	s+	s+	s+	—^f^	—	—	—	—	—	—	—
Stratton et al [50]	—	—	—	—	—	—	—	s+	ns^g^	ns	s+	—
Stratton et al [17]	s+	s+	s+	—	—	—	—	—	—	—	—	—
Carolan et al [36]	—	—	—	—	s+	—	—	—	—	—	s+	—
Xiong et al [57]	—	—	s+	—	—	—	—	—	—	—	—	—
Phillips et al [16]	m+^h^	s+	s+	s+	s+	m+	—	—	—	—	—	m+
Kuster et al [41]	~^i^ (1/2)^j^	—	—	—	—	—	—	—	—	—	—	—
Vertola et al [55]	+^k^ (9/9)	—	—	—	~ (5/6)	—	—	—	—	—	—	+ (3/3)
Thai et al [52]	+ (3/3)	+ (2/2)	+ (2/2)	—	—	—	—	—	—	—	—	—
López-Del-Hoyo et al [43]	~ (13/22)	~ (5/7)	~ (3/7)	+ (4/4)	—	—	~ (5/6)	—	—	—	—	~ (5/7)
Park et al [46]	~ (3/5)	-^l^ (0/1)	+ (1/1)	—	—	—	+ (1/1)	—	—	—	—	+ (1/1)
Velana et al [54]	+ (5/5)	+ (1/1)	+ (2/2)	+ (1/1)	—	—	—	—	—	—	—	—
Moe-Byrne et al [44]^m^	~ (3/4)	~ (2/6)	~ (2/6)	—	—	~ (1/2)	~ (2/3)	—	—	~ (3/4)	—	—
Jones et al [39]	~ (unclear)	+ (2/2)	—	—	~ (2/3)	—	—	—	—	—	—	—

^a^Sc: self-compassion and mindfulness.

^b^Pw: psychological well-being.

^c^En: employee engagement.

^d^In this study, the results for stress, anxiety, depression, and self-compassion are pooled together.

^e^In meta-analysis studies, s+ stands for small effect size (*g*<0.3).

^f^Not applicable.

^g^In meta-analysis studies, ns stands for nonsignificant results.

^h^In meta-analysis studies, m+ stands for medium effect size (0.3<*g*<0.5).

^i^In non–meta-analysis reviews, ~ stands for found conflicting evidence.

^j^(#/#) = (number of studies with significant evidence for this outcome) / (total number of studies reporting this outcome).

^k^In non–meta-analysis reviews, + stands for found unanimous evidence in favor of the effectiveness.

^l^In non-meta-analysis reviews, - stands for found no significant evidence.

^m^In this study, the results for anxiety and depression are reported together.

Depression was discussed in 7 reviews. Three meta-analyses [16,17,57] reported small but significant effects of DWPs on depression. Stratton et al [17] found an effect size of *g*=0.26 (95% CI 0.19-0.34, *k*=46, N=10,155, *I*²=67%); Phillips et al [16] reported an effect size of *g*=0.30 (95% CI 0.18-0.42, *k*=17, N=not available, *I*²=61%); and Xiong et al [57], specifically examining internet-based cognitive behavioral therapy programs, found an effect size of *g*=0.31 (95% CI 0.17-0.44, *k*=19, N=5898, *I*²=83%). In addition, 3 non–meta-analyses [46,52,54] reported significant findings regarding the impact of DWPs on depression. However, López-Del-Hoyo et al [43] found conflicting evidence on the effects of DWPs in this area.

Mindfulness and self-compassion outcomes were discussed in 1 meta-analysis by Phillips et al [16], which found a small effect size (*g*=0.42, 95% CI 0.24-0.60, *k*=5, N=not available, *I*²=0). In addition, 2 non–meta-analyses [43,54] reported significant evidence supporting the efficacy of DWPs in these outcomes.

Various secondary outcomes were also identified in the literature, including perceived well-being, psychological resilience, and sleep-related symptoms such as insomnia. For perceived well-being, 2 meta-analyses [16,36] reported small effect sizes. Carolan et al [36] found an effect size of *g*=0.37 (95% CI 0.23-0.50, *k*=21, N=2438, *I*^2^=81%), and Phillips et al [16] found a similar effect size of *g*=0.35 (95% CI 0.25-0.46, *k*=7, N=not available, *I*^2^=0). However, 2 non–meta-analyses [39,55] found conflicting evidence. For psychological resilience, 1 non–meta-analysis [44] found conflicting evidence in the literature. Regarding insomnia, 1 meta-analysis [16] reported a medium effect size (*g*=0.52, 95% CI 0.39-0.65, *k*=6, N=not available, *I*²=0%) after removing an outlier. In addition, 1 non–meta-analysis [44] found conflicting evidence on insomnia outcomes.

Interestingly, several reviews reported on job-related outcomes. One meta-analysis [50] focused exclusively on workplace-related outcomes, finding a small effect on employee engagement (*g*=0.19, 95% CI 0.10-0.28, *k*=10, N=2497, *I*²=16%) but no significant effect on absenteeism (*g*=0.28, 95% CI −0.02 to 0.57, *k*=12, N=3325, *I*²=94%) or presenteeism (*g*=0.18, 95% CI −0.05 to 0.41, *k*=8, N=2102, *I*²=85%). Regarding presenteeism, 1 non–meta-analysis [44] found conflicting evidence related to tailored DWPs. For employee productivity, 2 meta-analyses found small effect sizes: Stratton et al [50] reported *g*=0.16 (95% CI 0.03-0.29, *k*=16, N=3053, *I*²=62%), and Carolan et al [36] found *g*=0.25 (95% CI 0.09-0.41, *k*=13, N=1295, *I*²=75%). In terms of job burnout, Phillips et al [16] found a medium effect size (*g*=0.51, 95% CI 0.26-0.75, *k*=8, N=not available, *I*²=79%). Two non–meta-analyses [46,55] reported unanimous evidence supporting the efficacy of DWPs in reducing burnout, while López-Del-Hoyo et al [43] found conflicting evidence.

##### Acceptability

Two non–meta-analyses [36,47] investigated the acceptability of DWPs related to mental health (Table 4). According to these reviews, uptake rates—the proportion of targeted employees who start the program—are an underreported measure of acceptability in the existing literature. The average uptake rate is 11%, indicating that slightly more than 1 in 10 targeted employees initiate the program. Scheutzow et al [47] also identified several factors contributing to lower uptake rates, including employees’ fear of workplace stigmatization and their preference to keep health matters separate from workplace experiences. These barriers emphasize the need for organizations to address concerns about privacy and stigma to improve participation in DWPs.

**Table 4:** Acceptability of mental health digital wellness programs.

Study	Acceptability measures							
	Uptake	Adherence rate	Attrition rate	Dropout rate	Compliance rate	Satisfaction score	Int^a^	PU^b^
Scheutzow et al [47]	11% (n=2)	54% (n=2)	32% (n=3)	51% (range 15%-68%; n=7)	68% (n=2)	82% (n=10)	55% positive (n=9)	85%
Carolan et al [36]	—^c^	—	23% (range 3%-54%; n=20)	—	45% (range 3%-95%; n=19)	—	—	—

^a^Int: interest or willingness to use.

^b^PU: perceived usability or usefulness.

^c^Not applicable.

Regarding adherence rates, 2 empirical studies reported an average rate of 54%, as indicated by the study by Scheutzow et al [47]. However, the definition of adherence varies across these studies. One study defines adherence as continued participation after the first day of the intervention, while the other measures it as completing a specified number of activities within a given time frame, akin to a compliance rate. As a result, these findings should be interpreted with caution.

Attrition, dropout, and completion rates—which are similar measures—vary significantly across empirical studies. According to the literature reviews, the average rates for mental health DWPs are 32% for attrition, 51% for dropout, and 68% for completion according to the results in Table 4. The most frequently cited reasons for employees dropping out of these programs include concerns about privacy, lack of time, technical difficulties, lack of motivation, confidence in their ability to manage stress independently, dissatisfaction with the interventions, and high stress levels at the start of the program. In addition, the data suggest that younger employees are more likely to drop out of these interventions [47].

Higher completion rates are observed in DWPs delivered over shorter periods, those employing self-monitoring or tailored interventions, and those using secondary modalities such as email and SMS text messaging to enhance user engagement [36]. Finally, available evidence indicates that employees generally express high levels of satisfaction with DWPs, rating them as useful and showing a strong willingness to continue using them. However, Scheutzow et al [47] note that these positive satisfaction ratings often contrast with the unfavorable attrition and completion rates observed in many primary studies.

#### Physical Activity

##### Overview

The second most frequently discussed health domain is physical activity. According to the World Health Organization, physical activity refers to any movement of the human body, whether it involves performing daily tasks or altering one’s physical position [63]. Insufficient physical activity has long been associated with a range of health complications, including cancer, anxiety, depression, cardiovascular disease, musculoskeletal disorders, and premature death [64,65]. Organizations implement interventions in both sedentary workplaces and physically demanding environments to reduce health risk factors [66]. While sedentary behavior and physical activity interventions are sometimes discussed separately in the literature, we examine them together because both aim to increase active behavior. Table 5 summarizes the literature reviews within this domain. All the reviews in this health domain assess the efficacy of the programs. While most (7/9, 77%) examine the changes in intervention outcomes, 4 out of 9 (44%) examine the mechanisms of action used in these interventions. In addition, 1 review investigates the acceptability of these programs.

**Table 5:** Mapping of physical activity digital wellness programs.

Study	Type of RQ^a^	Digital technologies						Outcomes				
		Wb^b^	Ap^c^	Email	Ms^d^	Wearables	Am^e^		Sb^f^	An^g^	Presenteeism	Pw^h^
Jung and Cho [40]	Efficacy		✓		✓	✓	✓					
Aneni et al [18]	Efficacy	✓		✓			✓					
Freak-Poli et al [38]	Efficacy					✓	✓		✓	✓		✓
Thai et al [52]	Efficacy	✓	✓	✓		✓	✓					
Paganin and Simbula [45]	Efficacy		✓				✓		✓			
Sevic et al [48]	Efficacy and acceptability	✓	✓	✓		✓	✓		✓	✓	✓	
Buckingham et al [35]	Efficacy, mechanisms of action, and acceptability		✓			✓	✓		✓	✓	✓	✓
Tung et al [53]	Mechanisms of action	✓	✓			✓						
Damen et al [37]	Mechanisms of action	✓	✓	✓		✓						

^a^RQ: research question.

^b^Wb: websites and online telecommunication.

^c^Ap: apps and software.

^d^Ms: messaging services.

^e^Am: amount of physical activity (steps, duration, metabolic equivalent of task, etc).

^f^Sb: sedentary behavior (time, frequency of transitions, etc).

^g^An: anthropometric (weight, waist circumference, etc) and blood test indicators.

^h^Pw: psychological well-being.

##### Efficacy

The interventions in this health domain are less varied than those in the mental health domain, and the evidence on the efficacy of these interventions can be summarized into primary and secondary outcomes. As shown in Table 6, the primary outcomes of interest are measures such as the number of steps taken and the duration of physical activity, which we refer to as the amount of physical activity. A meta-analysis conducted by Jung and Cho [40] found that DWPs have a small but significant effect on the amount of physical activity (*g*=0.22, 95% CI 0.03-0.41, *k*=8, N=not available, *I*²=78%). However, 5 non–meta-analyses [18,35,38,48,52] reported conflicting evidence, with roughly half of the empirical studies finding significant changes in the amount of physical activity.

The secondary outcomes include measures of sedentary behavior (eg, sitting time and frequency of transitions to standing), anthropometric measures (eg, weight and waist circumference), physical readiness (eg, maximal oxygen uptake and aerobic capacity tests), and psychological well-being. These outcomes have been less-frequently discussed in prior studies. However, some non–meta-analyses provide evidence that physical activity DWPs can be effective in improving these outcomes.

Regarding sedentary behavior, 3 non–meta-analyses [35,38,48] reported conflicting evidence, with approximately half of the empirical studies showing significant improvements. For anthropometric measures, 2 non–meta-analyses [35,38] reported conflicting evidence, whereas Sevic et al [48] found only nonsignificant results. In terms of physical readiness, Buckingham et al [35] identified 2 interventions with conflicting results, whereas Sevic et al [48] found only 1 study with nonsignificant results. Regarding psychological well-being, Freak-Poli et al [38] reported conflicting evidence, whereas Buckingham et al [35] found significant positive effects.

**Table 6:** Summary of physical activity digital wellness program outcomes.

Study^a^	Outcomes				
	Am^b^	Sb^c^	An^d^	PR^e^	PW^f^
Jung and Cho [40]	s+^g^	—^h^	—	—	—
Aneni et al [18]	~^i^ (3/11)^j^	—	—	—	—
Freak-Poli et al [38]	~ (4/10)	~ (1/2)	~ (4/14)	—	~ (1/2)
Thai et al [52]	~ (3/4)	—	—	—	—
Sevic et al [48]	~ (3/7)	~ (2/3)	-^k^ (0/3)	- (0/1)	—
Buckingham et al [35]	~ (14/25)	~ (4/10)	~ (3/5)	~ (1/2)	+^l^ (2/2)

^a^We excluded the study by Paganin and Simbula [45] because results were not presented in line with our categories.

^b^Am: amount of physical activity (steps, duration, metabolic equivalent of task, etc).

^c^Sb: sedentary behavior (time, frequency of transitions, etc).

^d^An: anthropometric measures (weight, waist circumference, etc).

^e^PR: physical readiness (maximal oxygen uptake and aerobic readiness).

^f^PW: psychological well-being.

^g^In meta-analysis studies, s+ denotes small effect size (*g*<0.3).

^h^Not available.

^i^In non–meta-analysis studies, ~ denotes conflicting evidence.

^j^(#/#) = (number of studies with significant evidence for this outcome) / (total number of studies reporting this outcome).

^k^In non-meta-analysis reviews, - stands for found no significant evidence.

^l^In non–meta-analysis reviews, + stands for found unanimous evidence in favor of the effectiveness.

##### Mechanisms of Action

Four non–meta-analyses [35,37,48,53] examined mechanisms of action. These studies mainly conducted a scoping survey of the literature on DWPs with two objectives: (1) categorizing the intervention into existing taxonomies and (2) summarizing the theories used in the development of DWPs. These non–meta-analysis reviews are summarized in subsequent paragraphs.

Three reviews [37,48,53] categorized physical activity DWPs into the established taxonomies of behavior change techniques and intervention strategies [67,68]. According to these taxonomies, behavior change techniques are specific activities used in interventions to induce a change in one’s behavior, whereas intervention strategies (also known as intervention functions) are high-level approaches to design interventions. Sevic et al [48] and Tung et al [53] examined the scope of behavior change techniques and intervention strategies used in DWPs. According to these reviews, enablement and education are the most frequent intervention strategies. Enablement refers to those intervention strategies that enhance capabilities and opportunities to change behavior. It can be implemented through various behavior change techniques such as self-monitoring, feedback provision, and goal setting and action planning. For its part, education refers to the techniques that increase one’s knowledge of health-related topics. It can be implemented through various behavior change techniques such as providing instructions for healthy behavior and informing of the consequences of behavior. These reviews state that they could not clarify which behavioral techniques and strategies are more successful than others due to the heterogeneous characteristics of interventions compared with the limited number of empirical studies in the literature.

The fourth non–meta-analysis review [35] presents the theories used in the development of physical activity DWPs. In this regard, the most frequently cited theories are socioecologic theory, self-determination theory, social cognitive theory, and the theory of reasoned action. Similarly, this review does not discuss which theories are more successful in designing DWPs. Three reviews in our sample [37,45,53] agree that most empirical studies lack a theoretical basis for developing DWPs in this domain.

##### Acceptability

The acceptability of physical health DWPs is discussed in one literature review, which focuses on mHealth technologies [35]. Three main acceptability outcomes are reported in the surveyed empirical studies. The most commonly reported outcome is the attrition rate of interventions, which refers to the number of participants who fail to provide data at the final follow-up. The average attrition rate is 18%, which is considered acceptable for health promotion interventions. However, some interventions report attrition rates as high as 74%, indicating significant variability in the acceptability of the programs. This variability is attributed to several factors including employee demographics (with higher attrition rates among females and younger employees) and the duration of the interventions (with higher attrition in interventions lasting more than 12 weeks). Buckingham et al [35] also cite possible reasons for the lack of engagement after initial use, including privacy concerns, diminished interest, and lack of trust in the accuracy of the technologies. Despite the wide range of attrition rates, Buckingham et al [35] report that employees who continued using the programs until the follow-up generally expressed high levels of satisfaction. However, due to the high heterogeneity of interventions, the review was unable to provide a clear explanation for the variability in attrition rates or the reasons for the decline in the use of technological components.

#### Weight Management

##### Overview

Weight management interventions aim to help individuals achieve and maintain a healthy body weight, which can reduce the risk of chronic diseases and improve overall health and quality of life. Compared with employees with normal weight, employees with overweight and obesity incur higher direct costs (eg, insurance claims) and indirect costs (eg, absenteeism) for their companies. It is estimated that organizational costs associated with employees with overweight and obesity are 11% and 45% higher, respectively, than those for employees with normal weight [69]. Weight management interventions typically involve a combination of strategies such as modifying dietary intake and increasing physical activity [70]. We identified 5 literature reviews on weight management DWPs (Table 7). These DWPs adopt either a universal approach targeting all employees or a targeted approach focusing specifically on employees with obesity. Only 1 review [42] focuses solely on the targeted approach.

**Table 7:** Mapping of reviews on weight management digital wellness programs.

Study	Type of RQ^a^	Digital technologies						Outcomes			
		Wb^b^	Ap^c^	Email	Ms^d^	Wearables	An^e^		Am^f^	D^g^	PR^h^
Jung and Cho [40]	Efficacy				✓		✓				
Aneni et al [18]	Efficacy	✓		✓	✓		✓		✓	✓	✓
Lee et al [42]	Efficacy	✓	✓	✓	✓	✓	✓				
Thai et al [52]	Efficacy	✓		✓	✓	✓	✓				
Tung et al [53]	Mechanisms of action	✓	✓		✓						

^a^RQ: research question.

^b^Wb: websites and online telecommunication.

^c^Ap: apps and software.

^d^Ms: messaging services.

^e^An: anthropometric measures (BMI, weight, waist circumference, etc).

^f^Am: amount of physical activity (steps, duration, metabolic equivalent of task, etc).

^g^D: dietary intake.

^h^PR: physical readiness (maximal oxygen uptake and aerobic readiness).

##### Efficacy

Table 8 summarizes the outcomes reported for weight management DWPs. As expected, the most frequently discussed outcomes are related to anthropometric measures such as BMI, weight, and waist circumference. The only meta-analysis [16] conducted in this domain failed to establish significant support for the efficacy of DWPs on these measures (*g*=0.02, 95% CI −0.07 to 0.10, *k*=4, N=not available, *I*²=0%). However, several empirical studies reported in non–meta-analyses show a significant effect of DWPs on anthropometric outcomes. Specifically, 2 non–meta-analyses [18,52] found evidence from high-quality studies indicating small to modest reductions in weight and waist circumference.

Furthermore, 1 non–meta-analysis review [42] found unanimous evidence supporting the effectiveness of weight management DWPs that adopt a targeted approach. These interventions used various strategies such as teleconsultation, health data monitoring, personalized advice, and the provision of online resources. This review highlights the notably high success rates of targeted weight management DWPs, a valuable finding given that obese employees tend to have higher absenteeism, greater health risks, and increased health care costs [69,71].

Secondary outcomes of weight management DWPs include the amount of physical activity, dietary intake, and physical readiness (eg, maximal oxygen uptake and aerobic capacity). Only 1 non–meta-analysis [18] included these secondary outcomes but did not find significant changes. This lack of evidence has been attributed to the limited number of empirical studies reporting on these secondary outcomes.

**Table 8:** Summary of weight management digital wellness program outcomes.

Study	Outcomes			
	An^a^	Am^b^	D^c^	PR^d^
Jung and Cho [40]	ns^e^	—^f^	—	—
Aneni et al [18]	~^g^ (2/6)^h^	-^i^ (0/3)	- (0/2)	- (0/1)
Lee et al [42]	+^j^ (11/11)	—	—	—
Thai et al [52]	~ (3/4)	—	—	—

^a^An: anthropometric measures (BMI, weight, waist circumference, etc) and blood test indicators (cholesterol, blood pressure, etc).

^b^Am: amount of physical activity (steps, duration, metabolic equivalent of task, etc).

^c^D: dietary intake.

^d^PR: physical readiness (maximal oxygen uptake and aerobic readiness).

^e^In meta-analysis studies, ns denotes nonsignificant results.

^f^Not available.

^g^In non–meta-analysis studies, ~ denotes found conflicting evidence.

^h^(#/#) = (number of studies with significant evidence for this outcome) / (total number of studies reporting this outcome).

^i^In non-meta-analysis reviews, - stands for found no significant evidence.

^j^In non–meta-analysis studies, + denotes found unanimous evidence in favor of the effectiveness.

##### Mechanisms of Action

One non–meta-analysis review [53] examines the mechanisms of action in weight management programs. This review highlights that digital technologies can support various behavioral change strategies commonly used in these programs, such as enablement and education. The enablement strategy includes behavior change techniques such as self-monitoring of sitting time, self-monitoring of dietary behavior, and telemonitoring of physical activity data by a remote coach. The education strategy involves techniques such as delivering instructive content about nutrition, health risks, and physical activity through various online channels. Although this literature review summarizes the range and frequency of these behavior change techniques, it is unable to identify which specific techniques are most effective due to the heterogeneity of the intervention characteristics.

#### Unhealthy Behavior Change

##### Overview

The DWPs in this category are designed to modify behavioral patterns associated with higher health risks, such as smoking, poor eating habits, and excessive alcohol consumption. These modifiable unhealthy behaviors are linked to the earlier onset of chronic diseases and can lead to avoidable costs for organizations, including increased absenteeism and health care expenses [72,73]. As shown in Table 9, we identified 4 non–meta-analysis reviews related to unhealthy behavior change; however, only one of these reviews [51] exclusively focuses on unhealthy behavior change as its primary topic of interest.

**Table 9:** Mapping of reviews on unhealthy behavioral change digital wellness programs.

Study	Type of RQ^a^	Digital technologies						Outcomes					Unhealthy behavior addressed
		Wb^b^	Ap^c^	Email	Ms^d^	Wearables	BC^e^		An^f^	MH^g^	WO^h^		
Aneni et al [18]	Efficacy	✓		✓			✓		✓			Diet and smoking	
Phillips et al [16]	Efficacy	✓					✓					Alcohol	
Sevic et al [48]	Mechanisms of action	✓					✓					Diet and alcohol	
Sundstrom et al [51]	Efficacy	✓		✓			✓			✓	✓	Alcohol (exclusively)	

^a^RQ: research question.

^b^Wb: websites and online telecommunication.

^c^Ap: apps and software.

^d^Ms: messaging services.

^e^BC: behavioral change (eg, change in dietary choices and frequency of drinking and smoking).

^f^An: anthropometric measures (BMI, weight, waist circumference, etc).

^g^MH: mental health (eg, stress, anxiety, and depression).

^h^WO: work-related outcomes (eg, time management on the job and rumination in the work).

##### Efficacy

Table 10 summarizes the findings for the 3 types of unhealthy behaviors targeted by DWPs: smoking, poor diet, and excessive alcohol consumption. The most frequently cited outcomes in these studies relate to changes in the occurrence of the targeted behaviors, such as the frequency of alcohol consumption and the amount of healthy food intake. All included non–meta-analyses [16,18,48,51] reported conflicting evidence regarding the efficacy of DWPs in reducing smoking, improving diet, and curbing alcohol consumption. It is important to note that all the diet interventions included in these reviews were a part of multiple-component health interventions that also incorporated physical activity or weight management modules. For their part, 2 non–meta-analyses evaluated secondary outcomes of behavioral change DWPs. Aneni et al [18] found conflicting evidence regarding the effectiveness of diet-focused DWPs, which showed effectiveness in reducing weight and blood pressure. Sundstrom et al [51] also found conflicting evidence on alcohol-related DWPs, specifically concerning their impact on employees’ mental health and work-life balance.

**Table 10:** Summary of unhealthy behavioral change digital wellness program outcomes.

Study		Outcomes		
		BC^a^	An^b^	MW^c^

**Diet**				
	Aneni et al [18]	~^d^ (6/7)^e^	~ (5/6)	—^f^
	Sevic et al [48]^g^	~ (3/4)	—	—
**Smoking**				
	Aneni et al [18]	~ (3/4)	—	—
**Alcohol**				
	Sevic et al [48]	+^h^ (2/2)	—	—
	Phillips et al [16]	~ (2/5)	—	—
	Sundstrom et al [51]	~ (4/7)	—	~ (4/5)

^a^BC: behavioral change (eg, change in dietary choices, frequency of drinking and smoking).

^b^An: anthropometrics (weight and blood pressure).

^c^MW: mental well-being (eg, stress, anxiety, and depression) and work-life balance (eg, work strain and time management).

^d^In non–meta-analysis studies, + denotes found unanimous evidence in favor of the effectiveness.

^e^(#/#) = (number of studies with significant evidence for this outcome) / (total number of studies reporting this outcome).

^f^Not available.

^g^The diet interventions included in this review were in a multicomponent health intervention.

^h^In non–meta-analysis studies, ~ denotes found conflicting evidence.

##### Mechanisms of Action

One non–meta-analysis review [48] outlines the behavior change techniques used in DWPs aimed at dietary improvements and alcohol reduction. According to this review, digital technologies facilitate several behavior change techniques, including prompting employees, providing feedback, enhancing self-belief, offering social support, giving instructions to support behavior change, introducing nudging objects to employees’ immediate environment, and raising awareness about the consequences of unhealthy behaviors.

#### Sleep Management

##### Overview

The DWPs in this health domain aim to improve sleep quality and, consequently, enhance daytime alertness and performance at work. Poor sleep quality among employees is associated with productivity losses, an increased risk of chronic conditions, slower and less-accurate cognitive responses, and a higher likelihood of fatal occupational accidents [74]. Sleep management is especially crucial for shift workers in stressful or high-risk occupations. As shown in Table 11, we identified 2 non–meta-analysis reviews [19,52] that focus on the efficacy of sleep management DWPs. Bullock et al [19] examine sleep management programs delivered via mobile apps specifically designed to help shift workers. Thai et al [52] focus on sleep management interventions implemented in organizations located in low- and middle-income countries.

**Table 11:** Mapping of reviews on sleep management digital wellness programs.

Study	Type of RQ^a^	Digital technologies						Outcomes	
		Wb^b^	Ap^c^	Email	Ms^d^	Wearables	SM^e^		SS^f^

Bullock et al [19]^g^	Efficacy		✓				✓		✓
Thai et al [52]	Efficacy		✓		✓		✓		✓

^a^RQ: research question.

^b^Wb: websites and online telecommunication.

^c^Ap: apps and software.

^d^Ms: messaging services.

^e^SM: sleep-related measures (sleep duration, sleep debt, sleep difficulties, etc).

^f^SS: symptoms of poor sleep (daytime fatigue and daytime sleepiness).

^g^This study is only limited to shift workers.

##### Efficacy

As shown in Table 12, the outcomes of sleep management DWPs are categorized into 2 main areas. The first category involves measures of sleep quantity and quality. Two non–meta-analysis reviews [19,52] found unanimous evidence supporting the effectiveness of DWPs in improving sleep-related outcomes. The second category pertains to symptoms of poor sleep, such as daytime fatigue and sleepiness. Similarly, both reviews found consistent evidence supporting the effectiveness of sleep management DWPs in alleviating these symptoms. Thai et al [52] suggest that sleep interventions that use digital technologies to provide tailored advice to employees are more effective than those offering on-demand content. However, both reviews caution that the body of evidence is still too small to draw definitive conclusions, and thus, the findings should be interpreted with care.

**Table 12:** Summary of sleep management digital wellness program outcomes.

Study	Outcomes	
	SM^a^	SS^b^
Bullock et al [19]	+^c^ (1/1)^d^	+ (1/1)
Thai et al [52]	+ (1/1)	+ (2/2)

^a^SM: sleep-related measures (eg, sleep duration, sleep debt, sleep difficulties).

^b^SS: symptoms of poor sleep (eg, daytime fatigue and daytime sleepiness)

^c^In non–meta-analysis studies, + denotes found unanimous evidence in favor of the effectiveness.

^d^(#/#) = (number of studies with significant evidence for this outcome) / (total number of studies reporting this outcome).

#### Other Health Domains

The final category includes interventions that do not fit into the previously discussed domains. Three of the identified reviews [45,48,52] only touch on these DWPs tangentially. Interventions in this category address diverse issues, such as health risk assessments, reducing risk factors for chronic diseases, improving workplace ergonomics, and lowering blood glucose levels in at-risk employees. Empirical studies referencing these interventions report significant outcomes. However, due to the heterogeneity of the health issues addressed and the limited empirical evidence available, we were unable to synthesize or aggregate the findings. As a result, we acknowledge their presence within this body of research without attempting further analysis.

## Discussion

In this section, we synthesize and critically analyze the findings on DWPs with a focus on understanding efficacy, durability, mechanisms of action, acceptability, and targeting approaches across the identified health domains. While the findings reveal promising outcomes in several areas, they also underscore significant limits and gaps in the literature that necessitate further investigation to fully harness the potential of these programs.

### Principal Findings

The findings on DWPs indicate that navigating this diverse body of evidence requires a detailed examination of various aspects of these programs, including their targeted health domain, their reported outcomes, and their recruitment strategies. Principal findings have been summarized according to these aspects.

First, this meta-review shows mixed or conflicting evidence regarding the efficacy of DWPs for mental health and physical activity, the two most studied domains. In the mental health domain, while some reviews highlight small to medium positive effects on outcomes such as stress, anxiety, and depression, others report conflicting or inconclusive findings. For example, while some interventions lead to meaningful reductions in stress and anxiety [16,17], these effects are not consistently observed across all reviews [43,46]. Similarly, for physical activity, the efficacy of DWPs varies significantly. Although several studies indicate increases in the amount of physical activity mainly through goal-setting and self-monitoring features (eg, [35,40]), others show limited impact. This suggests that factors such as intervention design and participant engagement may influence outcomes. Consequently, while DWPs hold potential for improving mental health and physical activity, the inconsistent evidence highlights the need for deeper exploration of the factors that make certain interventions more successful than others.

Second, in the domain of weight management, non–meta-analyses [18,42,52] report significant reductions in BMI and body weight, especially among individuals with obesity participating in DWPs [42]. These results suggest that targeted interventions aimed at high-risk populations are more effective than generic programs. Although non–meta-analyses report many interventions with significant improvements in BMI and body weight, the only meta-analysis study on weight management DWPs [40] failed to show a significant change. The lack of convergence between the non–meta-analyses and the meta-analysis study in this health domain suggests that factors such as health risk perception may influence intervention efficacy.

Third, the evidence for unhealthy behavioral change, such as smoking cessation and alcohol reduction, is promising but limited. While the available non–meta-analysis reviews [16,18,48,51] indicate significant reductions in unhealthy behaviors, there is a lack of sufficient studies to provide comparative insights. Thus, while DWPs appear effective for behavior change, the relative effectiveness of different interventions remains unclear due to the limited evidence base.

Fourth, non–meta-analysis reviews in the sleep management domain show promising effects of DWPs on sleep outcomes, such as duration, latency, and quality [19,52]. The agreement between studies highlights the efficacy of DWPs in improving sleep health, but the body of evidence is limited to a few studies.

Fifth, DWPs are generally well-accepted by participants, particularly when they are personalized, user friendly, and easily accessible. However, acceptability measures are not free from bias. For example, satisfaction surveys typically gather feedback only from participants who complete the program, making these results susceptible to survival bias. In other words, while high satisfaction scores may suggest the program’s success, they can inflate acceptability measures by excluding the opinions of dissatisfied users who dropped out. Similarly, compliance rates, often gauged through log-in data, may understate the program’s success if users disengage after achieving their desired outcomes. It is crucial for researchers to recognize and account for these limitations in their studies.

In addition, the findings of this meta-review emphasize that the effectiveness and acceptability of DWPs are influenced by how user friendly and personalized they are. This aligns with prior research [75] that indicates the effective use of self-monitoring technologies depends on users’ attitude toward the system and their perceived role in managing their conditions. In this respect, providing more guidance to reliant users (those who assume a passive role in managing their health) and greater autonomy to engaged users (those who assume an active role in managing their health) could enhance program success. This suggests customizing DWPs to individual user profiles can maximize benefits.

Finally, this meta-review highlights that the recruitment strategy of DWPs plays a crucial role in determining their efficacy. DWPs that use a targeted approach, recruiting participants based on risk factors or health conditions, tend to produce larger effect sizes than those with a universal approach. Review studies focusing on mental health, weight management, and unhealthy behavioral change demonstrate that targeted programs tend to yield better outcomes. In contrast, DWPs with a universal approach may produce smaller effect sizes, likely due to the dilution of outcomes across a more diverse participant pool. For example, individuals with moderate alcohol consumption may show minimal change, reducing the overall impact measured in the program [51]. Therefore, it is important to interpret the results of DWPs in light of their recruitment strategy and acknowledge that universal programs may not achieve the same level of effectiveness as targeted interventions.

### Gaps and Promising Avenues for Research

This study exposed critical gaps and highlighted areas where further research is needed. The meta-review approach allowed us to survey a rich body of research that focuses on the same phenomenon but progresses in parallel with their focus on different health domains. We identified several gaps that were shared across various health domains. However, some gaps were identified by borrowing ideas from the research in other health domains. For example, a lack of focus on mechanisms of action in the mental health domain became apparent after comparing reviews in this domain with the ones in other health domains. In what follows, we highlight areas with significant gaps and provide recommendations to guide future research. The most important area concerns the inconsistent findings that were observed for the two most studied domains: mental health and physical activity.

Regarding mental health, the inconsistent findings are linked primarily to outcomes such as stress, anxiety, and depression. To resolve this inconsistency by explaining when DWPs have beneficial effects, moderation analysis is useful. This type of analysis examines whether the effects of DWPs, such as their stress-reducing effects depend on certain moderating factors [76]. Since technology is at the core of DWPs, the literature on technostress provides several moderating factors that are worth exploring. In particular, self-efficacy is a relevant factor because it assesses the extent to which persons believe that they have the ability to use technologies such as DWPs successfully (eg, [77,78]). Usually, people with higher levels of self-efficacy are more motivated to use these technologies effectively and make a greater effort to learn about them, whereas individuals with lower levels of self-efficacy give up more easily when the benefits of using the digital technology do not accrue immediately. This suggests that most people with higher levels of self-efficacy will use DWPs more effectively and will derive greater mental health benefits from using them than those with lower levels of it. However, this might not always be the case as the success of a DWP likely depends on its design elements and content. Even expert users with high levels of self-efficacy might become frustrated or disengaged when DWPs are not well-designed or are not sufficiently user friendly. For example, poor navigation features, a clunky interface, or overly simplistic features might all hinder engagement with DWPs and thus frustrate users. This means that not all users with high levels of self-efficacy will derive greater mental health benefits from DWPs. Notwithstanding this fact, self-efficacy holds the potential to resolve some of the conflicting findings in the literature (eg, [79]).

Other moderating factors from the technostress literature are also relevant. Worker age is an important factor because older workers experience a greater mental workload when they engage with technologies such as DWPs [80-83]. Since this increased mental workload leads to stress [83], it can undermine the stress-reducing potential of DWPs. Other relevant moderating factors include differing levels of motivation to improve health-related outcomes, differing levels of IT support available to use DWPs, and differing levels of experience with such digital tools as messaging services, online telecommunication, and videoconferencing apps [78,83-86]. These factors can impact how effectively workers engage with DWPs and thus affect the degree to which the potential benefits of these technologies are realized. DWPs can be expected to lead to greater benefits when users are more motivated to improve their health when they receive more IT support, and when they have more experience with relevant tools. This means that these factors can help explain some of the conflicting findings in the extant literature.

However, the big 5 personality traits are likely the most promising moderating factors because they have been shown to moderate various relationships between engagement with technology and stress-related variables (eg, [87-89]). They have also been shown to affect the stress process more broadly [90], and they include 5 constructs: neuroticism, openness, agreeableness, extraversion, and conscientiousness. Regarding the moderating impact of personality traits on the stress-reducing potential of DWPs, individuals high in neuroticism are likely to report lower mental health benefits from engaging with DWPs. This is because they tend to experience greater dissatisfaction, frequently complain, and perceive even minor frustrations as overwhelming, making them less likely to find DWPs beneficial. In contrast, individuals high in openness are typically curious and willing to explore new experiences, which can lead to greater satisfaction with DWPs. Their openness to new approaches may result in more positive evaluations of the benefits of engaging with these systems, making them more likely to perceive DWPs as beneficial.

Regarding agreeableness, individuals high in this trait tend to be cooperative and are more likely to follow the advice of nurses, doctors, and artificial intelligence chatbots [87-89]. As a result, they are likely to derive greater benefits from their engagement with DWPs compared with those with lower agreeableness. Similarly, extraverted individuals are typically social, active, and outgoing. They often use technology strategically to maintain a positive social image, which suggests that they may view DWPs as a tool for sustaining well-being, job performance, and influence within the organization. Consequently, they are more likely to engage effectively with DWPs and perceive their engagement as beneficial. Finally, conscientious individuals tend to be detail oriented, dependable, and persistent. Even if they experience initial frustration, their intrinsic motivation and strong work ethic make them more likely to persist in using DWPs. Their commitment to efficiency and effectiveness further enhances their likelihood of successfully integrating DWPs into their routines.

Regarding physical activity, our study highlights that goal setting is a widely used behavior change technique. Goal-setting theory suggests several important moderators that influence its effectiveness. Specifically, for goal setting to be successful, individuals must possess both the ability and the situational resources necessary to achieve the goal [91]. In addition, goal importance and commitment to goal attainment serve as key moderators of effectiveness. However, one particularly noteworthy moderator is feedback, which plays a crucial role in goal progress. Regular feedback enables individuals to adjust their effort and refine their strategies, ensuring they stay on track toward goal attainment [91]. This suggests that the 2 primary drivers of increased physical activity in DWPs, goal-setting and self-monitoring features, may interact. Future research could explore whether the impact of goal setting on physical activity is enhanced when combined with self-monitoring features that provide a continuous stream of relevant feedback.

This meta-review also sheds light on methodological and structural attributes of the literature that future research should focus on. One such attribute across all health domains is the scarcity of long-term studies. Although many DWPs demonstrate initial effectiveness, the sustainability of these effects remains largely unexplored. For example, prior literature reviews on mental health and physical activity [17,35,38] have reported a decline in positive outcomes after a few months. However, the specific conditions under which these reductions occur, and whether they are more pronounced in DWPs compared with nondigital wellness programs, are still unclear. A recent study provides evidence of a temporary digital placebo effect in smartphone-based mental health interventions [92]. The digital placebo occurs when users experience benefits from digital technologies due to their beliefs in the tools rather than from their actual functionalities [93]. Initial expectancy and credibility of digital tools can taper off and potentially lead to reduced effectiveness in outcomes [92]. Yet, it is not clear who is more prone to this effect, what factors in intervention designs exacerbate it, and what practices can mitigate its impact. Hence, future research must prioritize longitudinal studies that answer these questions regarding DWPs and potentially compare them with their nondigital counterparts.

Another significant gap in the literature is the limited understanding of the mechanisms that drive the success of DWPs. Only 4 reviews in our sample have delved into the specific behavior change techniques that are used in these programs [35,37,48,53]. While these reviews offer valuable insights, many questions remain unanswered regarding the comparative advantage of various techniques. Surprisingly, despite the numerous reviews conducted on mental health DWPs, we did not find any review that examines the mechanisms of action used in this domain. Although some reviews [17,57] compare effect sizes of various therapeutic approaches such as cognitive behavioral therapy and positive psychology, it remains unclear whether their success is attributable to the therapeutic approaches or the effective activation of causal mechanisms of action by digital interventions. Psychotherapy scholars have recently called for the integration of these mechanisms into the research on mental health interventions [94-96]. This allows researchers to develop treatment strategies that focus on the most important mechanisms [96] and to examine moderating factors that influence active mechanisms of change [95].

Moreover, recent research underscores the importance of studying behavior change techniques in light of the causal mechanisms of change [97,98]. For example, Carey et al [98] provide an extensive list of causal mechanisms of action used in behavioral interventions. Future research on DWPs can leverage this list to examine how IT-related factors (eg, design and personal innovativeness with IT) and organization-related factors (eg, organizational culture and perceived organizational support) can weaken or strengthen the mechanisms of action applied in DWPs.

In a similar vein, prior reviews [35,48] call attention to the lack of strong theoretical foundations in prior research. We echo this concern and highlight 2 potential consequences that have created a gap in our understanding of DWPs. First, an atheoretical approach to designing health interventions limits our ability to generalize findings. For example, combining behavior change techniques can “lead to combinations of counteracting mechanisms in mHealth designs that obfuscate the techniques’ effects” [99]. Leveraging health and behavior change theories, researchers can articulate the boundary conditions of compatible interventions. Second, an atheoretical approach limits our ability to understand why and under what conditions DWPs influence organization-related outcomes such as burnout. For example, the theory of job demands-resources [100] provides a useful perspective in studying the organization-related outcomes of mental health DWPs. However, it should be further elaborated to explain how the characteristics of DWPs can contribute to job resources. To advance the field, it is essential that future studies are grounded in relevant theoretical frameworks, elucidate the processes by which DWPs influence health- and organization-related outcomes, and ultimately create value in organizations.

This meta-review also highlights limitations in the scope of interventions examined in the literature. While existing studies primarily focus on individual-level outcomes, such as personal health and productivity, wellness interventions can also be analyzed at higher organizational levels, including work groups and entire organizations [101]. Research on nondigital wellness initiatives has demonstrated organization-level outcomes, such as insurance claims and cost-benefit analyses of wellness programs [102]. However, similar outcomes have not yet been examined in the context of digital wellness initiatives. To address this critical research gap, future studies could leverage archival data, such as insurance claims and unit performance reports, to assess the broader organizational impact of DWPs.

As mentioned earlier, the variability in outcome measures across studies presents a significant barrier to synthesizing findings and drawing generalizable conclusions. Particularly in the domains of physical activity and mental health, there is a need for standardized outcome measures that can be consistently applied across studies. This would allow for more robust comparisons and a clearer understanding of the true impact of DWPs. Moreover, most studies focus on health-related outcomes neglecting the organizational implications of these programs. Therefore, there is a clear need for more research that links DWPs to positive organizational outcomes such as productivity, presenteeism, retention, and job satisfaction. Understanding these links is crucial for demonstrating the value of DWPs to employers and other stakeholders.

Finally, the role of technology in DWPs is often treated superficially, with scant attention given to how specific technological features influence the efficacy and acceptability of these programs. For example, engagement is an understudied factor in understanding the efficacy of DWPs beyond common measures of acceptability, such as frequency of use. Engagement in digital interventions is a multidimensional construct that involves physical, cognitive, and affective investment in the stimulus (eg, app) [103]. Research shows that using more features of wearable trackers (ie, a form of physical engagement) is associated with higher health-related outcomes [15]. Future research should demonstrate how and under what conditions each dimension of engagement contributes to intervention outcomes. Moreover, despite the large body of research on engagement with technology, there is limited understanding of how to enhance engagement in the technological stimulus in DWPs. For example, Nahum-Shani et al [103] suggest that environmental dynamics and participants’ information overload are important factors in the context of information-rich digital interventions. In this respect, future research should elucidate how employee-related factors (eg, cognitive and emotional resources) and organizational environment (eg, supportive climate) contribute to the engagement and eventually to the success of DWPs.

In addition, abstracting the technology components of DWPs into a device or mobile app without considering the underlying function of technology downplays its contribution to the success of DWPs. For example, a mobile app can have various functions such as delivering nudges and facilitating self-monitoring. The reviews in our corpus did not present their findings in this aspect, suggesting that accumulated evidence is lacking regarding the role of technology in DWPs. Future research should delve deeper into the technological aspects of DWPs by examining how different features and components of digital tools contribute to or detract from the success of digital interventions.

### Implications for Practice

The findings of this meta-review hold significant implications for organizations, particularly for human resource (HR) managers tasked with improving employee health and well-being. While DWPs show potential in promoting employee wellness, their effectiveness is not consistent across all health domains. The mixed evidence on mental health and physical activity—the two most studied areas—suggests that HR managers should have measured expectations regarding the immediate impact of these programs. Although DWPs can positively influence outcomes such as stress, anxiety, and physical activity, their success depends largely on program design, employee engagement, and the specific context of implementation. Therefore, HR managers should focus on tailoring DWPs to address the diverse needs of their workforce, ensuring interventions are personalized and adaptable. Conducting employee wellness assessments (eg, anonymous surveys, biometric screenings, or focus groups) can help organizations identify key health risks, understand employee preferences, and gauge engagement levels, allowing them to tailor interventions accordingly. By leveraging data-driven insights, HR managers can design more personalized and adaptable programs, ensuring that DWPs are aligned with employees’ specific needs rather than taking a one-size-fits-all approach. In addition, these assessments provide valuable feedback loops, enabling organizations to refine and optimize their wellness strategies over time, ultimately improving program effectiveness and employee well-being.

Given the variability in outcomes, regular monitoring and assessment are essential to ensure that DWPs remain relevant and impactful. HR managers should evaluate the programs over time by seeking employee feedback (eg, satisfaction) and monitoring various acceptability measures (eg, uptake rate, completion rate, and engagement) and effectiveness measures (eg, individual and organization-related outcomes). Relying solely on any one of these measures can provide a narrow perspective on the success of the programs. Implementing regular monitoring mechanisms, including A/B testing of intervention approaches, can help refine these initiatives for greater impact.

While DWPs have shown promise in facilitating behavioral changes such as smoking cessation and alcohol reduction, the evidence remains limited. HR managers should not expect immediate or significant results in these areas. To enhance the impact of DWPs on behavior change, these programs should be integrated with broader organizational strategies such as health coaching, peer support groups, and incentives for healthy habits. Creating a supportive culture that encourages ongoing healthy lifestyle choices can help sustain these behavioral changes over time. Organizations should embed digital wellness check-ins into daily workflows and encourage leadership to model healthy behaviors, such as setting boundaries for after-hours emails.

Technology plays a crucial role in the success of DWPs. Wearables, mobile apps, and online platforms have proven effective in promoting the health of employees in various domains. They enable many opportunities for implementing organizational interventions that can easily be scaled up. However, as reflected in the acceptability measures discussed in the literature, HR managers should acknowledge that there is a great deal of variation in terms of adoption, continuous use, and the extent of engagement with technological initiatives among employees. Examining the compatibility of technologies with the target population before implementing DWPs can contribute to the success of such programs. For example, if the target population of a weight reduction DWP is employees with obesity, the organization can assess their self-management style [75] before investing in those programs that highly depend on self-monitoring devices. Personalization of wellness plans, offering a mix of mobile apps, virtual coaching, and wearable devices, can further improve engagement by catering to diverse employee preferences.

For DWPs to be truly impactful, they must be part of a larger organizational culture that prioritizes employee well-being. HR managers play a pivotal role in promoting this culture by embedding wellness as a core organizational value. This involves not only offering DWPs but also aligning organizational policies with initiatives that support work-life balance, stress management, and mental health. A culture of shared responsibility for wellness can significantly enhance the effectiveness of DWPs, leading to more positive outcomes for both employees and the organization.

In addition to improving employee health, HR managers can consider the return on investment for the organization. Evidence from this meta-review suggests that DWPs can potentially impact organizational outcomes such as engagement, absenteeism, and productivity, particularly when addressing prevalent issues such as mental health, sedentarism, and weight management. Highlighting potential cost savings and productivity gains can help HR managers present DWPs as both a health initiative and a strategic investment, securing the necessary resources and support for successful implementation.

In conclusion, DWPs offer organizations a valuable opportunity to improve employee health, enhance productivity, and reduce health care costs. However, the success of these programs depends on thoughtful implementation, continuous evaluation, and a supportive organizational culture. HR managers are at the forefront of this effort, playing a critical role in designing, promoting, and sustaining effective DWPs that meet the diverse needs of their workforce. By leveraging cross-disciplinary expertise and integrating digital health interventions within existing workplace structures, organizations can maximize the potential of DWPs for long-term employee well-being and organizational success.

### Study Limitations and Future Research

This meta-review has several limitations. First, the initial abstract screening was conducted by a single reviewer, which may have introduced some selection bias. However, to minimize this risk, the reviewer applied a conservative approach, excluding studies only when it was certain they did not meet the inclusion criteria. Any study with uncertainty was flagged and discussed in consensus meetings among the authors before a final decision was made. While this approach helped mitigate bias, future meta-reviews may benefit from a dual-independent screening process to further enhance reliability. Second, the quality and scope of the included reviews varied, which could have affected the generalizability of our findings. Finally, while our review methodology was efficient, it may have missed recent studies due to publication delays. Future research should address these limitations by incorporating a broader range of studies and tackling more specific RQs inspired by this meta-review. In addition, there is a need for more rigorous methodological approaches, particularly in examining long-term efficacy, mechanisms of action, and the role of technology in DWPs. Researchers should also prioritize the development of standardized outcome measures and further explore the organizational implications of DWPs, which remain underrepresented in the literature.

### Conclusions

This meta-review provides a comprehensive synthesis of DWPs across various health domains, offering valuable insights into their efficacy and acceptability. The findings highlight promising outcomes, particularly in the areas of mental health, physical activity, and weight management, where interventions typically led to small to medium improvements. However, some inconsistencies persist in the extant literature that future studies must address. This study also reveals variability in efficacy depending on recruitment strategies, with targeted DWPs generally producing more substantial effects compared with universal programs. Significant gaps also remain, especially the scarcity of long-term studies and the limited understanding of the mechanisms driving DWP success. It also emphasizes the need for theory-driven research to better explain how and why certain DWPs are effective. For HR managers and organizations, the findings suggest that implementing tailored DWPs can enhance employee well-being and organizational outcomes. However, ongoing evaluation and adaptation are crucial to ensure these programs achieve their intended goals. In summary, while DWPs show great potential for improving employees’ wellness, further research is necessary to optimize their design and implementation to ensure they deliver sustainable benefits in diverse workplace settings.

## Appendix

Multimedia Appendix 1 PRISMA-ScR (Preferred Reporting Items for Systematic Reviews and Meta-Analyses for Scoping Reviews) checklist.

Multimedia Appendix 2 Full search strings.

## Abbreviations

DWP: digital wellness program
HR: human resource
PRISMA: Preferred Reporting Items for Systematic Reviews and Meta-Analyses
RQ: research question
