# Supplementary material for: Digital Wellness Programs in the Workplace: Meta-Review
Source: J Med Internet Res. 2025 Mar 14;27:e70982. doi: 10.2196/70982 (PMC11953596; doi:10.2196/70982)
Supplement: Multimedia Appendix 2 [file jmir_v27i1e70982_app2.docx]

**Search string: Web of Science**

((AB=("Systematic review") OR AB=("Scoping review") OR AB=("Narrative review") OR AB=("Descriptive review") OR AB=("Meta-analysis") OR AB=("Mapping review") OR AB=("Realist review") OR AB=("Meta-synthesis") OR AB=("Comparative review") OR AB=("PRISMA") OR AB=("conceptual review") OR AB=("literature review") OR AB=("Integrative review") OR AB=("meta-ethnography") OR AB=("meta-synthesis") OR AB=("Meta review") OR AB=("Meta-review") OR AB=("narrative review") OR AB=("narrative synthesis") OR AB=("qualitative review")) AND (AB=("Workplace") OR AB=("Work place") OR AB=("Employee") OR AB=("Occupational setting") OR AB=("Worker") OR AB=("Workforce") OR AB=("Organisational setting") OR AB=("Organizational setting") OR AB=("staff")) AND (AB=("m-health") OR AB=("mhealth") OR AB=("mobile health") OR AB=("e-health") OR AB=("ehealth") OR AB=("digital health") OR AB=("digital wellness") OR AB=("virtual care ") OR AB=("Self-Tracking") OR AB=("self quantification") OR AB=("self-quantification") OR AB=("Quantified self") OR AB=("wearable") OR AB=("iCBT"))) OR ((AB=("Systematic review") OR AB=("Scoping review") OR AB=("Narrative review") OR AB=("Descriptive review") OR AB=("Meta-analysis") OR AB=("Mapping review") OR AB=("Realist review") OR AB=("Meta-synthesis") OR AB=("Comparative review") OR AB=("PRISMA") OR AB=("conceptual review") OR AB=("literature review") OR AB=("Integrative review") OR AB=("meta-ethnography") OR AB=("meta-synthesis") OR AB=("Meta review") OR AB=("Meta-review") OR AB=("narrative review") OR AB=("narrative synthesis") OR AB=("qualitative review")) AND (AB=("Workplace") OR AB=("Work place") OR AB=("Employee") OR AB=("Occupational setting") OR AB=("Worker") OR AB=("Workforce") OR AB=("Organisational setting") OR AB=("Organizational setting") OR AB=("staff")) AND (AB=("physical health") OR AB=("physical activity") OR AB=("mental health") OR AB=("sleep health") OR AB=("psychological health") OR AB=("well-being") OR AB=("wellbeing") OR AB=("prescriptions") OR AB=("preventive health") OR AB=("sleep health") OR AB=("sleep disorders")) AND (AB=("App") OR AB=("information technology") OR AB=("information system") OR AB=("Digital") OR AB=("Platform") OR AB=("self-quantification") OR AB=("quantified self") OR AB=("Smartphone") OR AB=("internet-based") OR AB=("mobile phone") OR AB=("Cell phone") OR AB=("Virtual") OR AB=("teleconsultation")))

**Search string: ProQuest**

((ABSTRACT("Systematic review") OR ABSTRACT("Scoping review") OR ABSTRACT("Narrative review") OR ABSTRACT("Descriptive review") OR ABSTRACT("Meta-analysis") OR ABSTRACT("Mapping review") OR ABSTRACT("Realist review") OR ABSTRACT("Meta-synthesis") OR ABSTRACT("Comparative review") OR ABSTRACT("PRISMA") OR ABSTRACT("conceptual review") OR ABSTRACT("literature review") OR ABSTRACT("Integrative review") OR ABSTRACT("meta-ethnography") OR ABSTRACT("meta-synthesis") OR ABSTRACT("Meta review") OR ABSTRACT("Meta-review") OR ABSTRACT("narrative review") OR ABSTRACT("narrative synthesis") OR ABSTRACT("qualitative review")) AND (ABSTRACT("Workplace") OR ABSTRACT("Work place") OR ABSTRACT("Employee") OR ABSTRACT("Occupational setting") OR ABSTRACT("Worker") OR ABSTRACT("Workforce") OR ABSTRACT("Organisational setting") OR ABSTRACT("Organizational setting") OR ABSTRACT("staff")) AND (ABSTRACT("m-health") OR ABSTRACT("mhealth") OR ABSTRACT("mobile health") OR ABSTRACT("e-health") OR ABSTRACT("ehealth") OR ABSTRACT("digital health") OR ABSTRACT("digital wellness") OR ABSTRACT("virtual care ") OR ABSTRACT("Self-Tracking") OR ABSTRACT("self quantification") OR ABSTRACT("self-quantification") OR ABSTRACT("Quantified self") OR ABSTRACT("wearable") OR ABSTRACT("iCBT"))) OR ((ABSTRACT("Systematic review") OR ABSTRACT("Scoping review") OR ABSTRACT("Narrative review") OR ABSTRACT("Descriptive review") OR ABSTRACT("Meta-analysis") OR ABSTRACT("Mapping review") OR ABSTRACT("Realist review") OR ABSTRACT("Meta-synthesis") OR ABSTRACT("Comparative review") OR ABSTRACT("PRISMA") OR ABSTRACT("conceptual review") OR ABSTRACT("literature review") OR ABSTRACT("Integrative review") OR ABSTRACT("meta-ethnography") OR ABSTRACT("meta-synthesis") OR ABSTRACT("Meta review") OR ABSTRACT("Meta-review") OR ABSTRACT("narrative review") OR ABSTRACT("narrative synthesis") OR ABSTRACT("qualitative review")) AND (ABSTRACT("Workplace") OR ABSTRACT("Work place") OR ABSTRACT("Employee") OR ABSTRACT("Occupational setting") OR ABSTRACT("Worker") OR ABSTRACT("Workforce") OR ABSTRACT("Organisational setting") OR ABSTRACT("Organizational setting") OR ABSTRACT("staff")) AND (ABSTRACT("physical health") OR ABSTRACT("physical activity") OR ABSTRACT("mental health") OR ABSTRACT("sleep health") OR ABSTRACT("psychological health") OR ABSTRACT("well-being") OR ABSTRACT("wellbeing") OR ABSTRACT("prescriptions") OR ABSTRACT("preventive health") OR ABSTRACT("sleep health") OR ABSTRACT("sleep disorders")) AND (ABSTRACT("App") OR ABSTRACT("information technology") OR ABSTRACT("information system") OR ABSTRACT("Digital") OR ABSTRACT("Platform") OR ABSTRACT("self-quantification") OR ABSTRACT("quantified self") OR ABSTRACT("Smartphone") OR ABSTRACT("internet-based") OR ABSTRACT("mobile phone") OR ABSTRACT("Cell phone") OR ABSTRACT("Virtual") OR ABSTRACT("teleconsultation")))

**Search string: PsychInfo and Medline**

(("Systematic review" OR "Scoping review" OR "Narrative review" OR "Descriptive review" OR "Meta-analysis" OR "Mapping review" OR "Realist review" OR "Meta-synthesis" OR "Comparative review" OR "PRISMA" OR "conceptual review" OR "literature review" OR "Integrative review" OR "meta-ethnography" OR "meta-synthesis" OR "Meta review" OR "Meta-review" OR "narrative review" OR "narrative synthesis" OR "qualitative review") AND ("Workplace" OR "Work place" OR "Employee" OR "Occupational setting" OR "Worker" OR "Workforce" OR "Organisational setting" OR "Organizational setting" OR "staff") AND ("m-health" OR "mhealth" OR "mobile health" OR "e-health" OR "ehealth" OR "digital health" OR "digital wellness" OR "virtual care " OR "Self-Tracking" OR "self quantification" OR "self-quantification" OR "Quantified self" OR "wearable" OR "iCBT")) OR (("Systematic review" OR "Scoping review" OR "Narrative review" OR "Descriptive review" OR "Meta-analysis" OR "Mapping review" OR "Realist review" OR "Meta-synthesis" OR "Comparative review" OR "PRISMA" OR "conceptual review" OR "literature review" OR "Integrative review" OR "meta-ethnography" OR "meta-synthesis" OR "Meta review" OR "Meta-review" OR "narrative review" OR "narrative synthesis" OR "qualitative review") AND ("Workplace" OR "Work place" OR "Employee" OR "Occupational setting" OR "Worker" OR "Workforce" OR "Organisational setting" OR "Organizational setting" OR "staff") AND ("physical health" OR "physical activity" OR "mental health" OR "sleep health" OR "psychological health" OR "well-being" OR "wellbeing" OR "prescriptions" OR "preventive health" OR "sleep health" OR "sleep disorders") AND ("App" OR "information technology" OR "information system" OR "Digital" OR "Platform" OR "self-quantification" OR "quantified self" OR "Smartphone" OR "internet-based" OR "mobile phone" OR "Cell phone" OR "Virtual" OR "teleconsultation"))
